# Estimation of Human Body Vital Signs Based on 60 GHz Doppler Radar Using a Bound-Constrained Optimization Algorithm

**DOI:** 10.3390/s18072254

**Published:** 2018-07-12

**Authors:** Ting Zhang, Julien Sarrazin, Guido Valerio, Dan Istrate

**Affiliations:** 1 Zhejiang Provincial Key Laboratory of Information Processing, Communication and Networking (IPCAN), College of Information Science and Electronic Engineering (ISEE), Zhejiang University, Hangzhou 310027, China; 2 Sorbonne Universités, UR2, L2E, F-75005 Paris, France; julien.sarrazin@sorbonne-universite.fr (J.S.); guido.valerio@sorbonne-universite.fr (G.V.); 3 Sorbonne University, UTC CNRS UMR 7338, Biomechanics and Bioengineering (BMBI), University of Technology of Compiègne, BP 20529, Rue Personne de Roberval, 60205 Compiègne, France

**Keywords:** 60 GHz Doppler radar, bound-constrained optimization algorithm, breathing and heartbeat rate detection

## Abstract

In this study, a bound-constrained optimization algorithm is applied for estimating physiological data (pulse and breathing rate) of human body using 60 GHz Doppler radar, by detecting displacements induced by breathing and the heartbeat of a human subject. The influence of mutual phasing between the two movements is analyzed in a theoretical framework and the application of optimization algorithms is proved to be able to accurately detect both breathing and heartbeat rates, despite intermodulation effects between them. Different optimization procedures are compared and shown to be more robust to receiver noise and artifacts of random body motion than a direct spectrum analysis. In case of a large-scale constrained bound, a parallel optimization procedure executed in subranges is proposed to realize accurate detection in a reduced span of time.

## 1. Introduction

Patient telemonitoring is a good solution to help manage medical environments such as nursing homes and hospitals in daily tasks as well as patient managing, health monitoring, abnormality- and distress-situation detection [1], and activities of daily living recognition [2]. It can increase the quality of care and the efficiency of services provided. Indeed, it should facilitate daily tasks of caregivers in the cases of casual and continuous monitoring of chronic patients, elderly and dependent people. Several patient telemonitoring systems using different kinds of sensors have been proposed in the literature. A multicamera motion-capture system is proposed in [3], aiming at providing caregivers with timely access to the patient’s health status through mobile communication devices. In [4], a Distress Sound Extraction System for Elder Care was proposed. A fall detection system is presented in [5], based on smartphone accelerometer sensors using machine-learning classification algorithms. A prototype for remote healthcare monitoring in [6] uses wireless sensor network (WSN) pulse oximeters, environmental sensors and streaming video to monitor patients.

Large-sized sensors, such as cameras, microphones, oximeters and pulse sensors, could be intrusive for most of the monitored people. To this aim, telemonitoring systems have been giving increasing attention to the utilization of less-intrusive sensors such as pyroelectric infrared movement sensors. In this context, a monitoring system was developed in [7] to monitor patient activities of daily living, such as mobility, agitation, repartitions of stays, and displacements. A new benchmark for human activity-recognition algorithms was proposed based on infrared sensors [8]. A soft tracking system was proposed in [9] using an infrared ceiling sensor network and a novel algorithm for tracking multiple people. Indoor vital-signs telemonitoring can also provide a less-intrusive way to detect emergency situations, which is generally realized by Doppler radar systems. This noncontact technique [10,11] is very convenient for monitoring elderly and dependent people, as no sensor attached to the human body is required. Owing to the Doppler effect, the body displacements induced by physiological movements, such as heartbeat and breathing, can be detected by measuring phase shifts of the reflected radar signal.

With this technique, detecting respiration and heartbeat rates simultaneously can be achieved by transmitting a monochromatic wave towards the person whose vital signs are to be estimated. The reflected wave is then received and analyzed. However, particular distances between the radar and the person can lead to a null received signal. In order to avoid this null-point detection problem, one can work at double sideband frequencies [12] or use an in-phase quadrature (IQ) receiver for demodulation [13], where the local oscillator (LO) signal is split into two chains with π/2 phase difference, ensuring that at least one output would not be trapped into the null point. The latter is widely used in Doppler radar systems. Two different demodulation techniques with IQ receiver are possible: arctangent demodulation [14] and complex demodulation [15]. In [16], the reflected baseband signal after the arctangent demodulation is firstly processed through a wavelet filter to separate the heartbeat signal from the respiration. An ensemble empirical mode decomposition (EEMD)-based algorithm is then applied to extract the heartbeat rate. The arctangent demodulation is straightforward to realize; nevertheless, this technique is sensitive to the DC offset caused by hardware imperfections [17] and necessitates the preprocessing Gram–Schmidt procedure for compensating the IQ mismatch [18]. The complex demodulation can avoid these drawbacks but undesired intermodulation and harmonic components are present. An important limitation of these components is the difficulty in determining the fundamental component from merged sinusoidal components. It was shown that the carrier frequency of a continuous-wave (CW) Doppler radar could be limited to the lower region of the Ka-band to decrease possible intermodulation effects between the two movements [19]. Nevertheless, three different carrier frequencies are compared in [20], and measured results show that operating at higher frequencies leads to a more accurate detection of heartbeat rate. Therefore, a 60 GHz Doppler was developed in [21] to detect weak heartbeat signal, whereas the intermodulation effect of the baseband signal is more important. Moreover, if the harmonic of respiration is very close to the fundamental of the heartbeat, it becomes impossible to distinguish the contributions. This phenomenon, referred to as ‘ambiguity’ in this paper, should be carefully addressed. In all cases, a direct peak detection of the spectrum of reflected baseband signal is consequently not reliable any more; this requires a spectral estimation algorithm for a robust determination of the frequency components. In [22,23], a harmonic-path algorithm was developed to determine both heartbeat and respiration rates by taking into account all harmonic components of the whole spectrum, but the ambiguity problem is not addressed. A RELAX algorithm was used in [24] for the spectrum estimation with a 20 GHz Doppler radar, which is based on the minimization of a nonlinear least-square fitting problem. The heartbeat and respiration rates are estimated by recognizing all sinusoidal components in the spectrum, but the intermodulation effects between them is not considered.

In addition to the ambiguity, the presence of random body movement can interrupt the detection of small physiological signals and should be eliminated for accurate sensing. A specific model for this movement, such as a sinusoidally modulated Gaussian signal, is considered and compensated for by performing empirical mode decomposition in [25], under the assumption that the movement waveform has a broader frequency band than desired physiological movements. For other possible types of movements, multiple transceivers can be used to perform the measurement of different sides of the body [15]. Another solution is to operate with two or more frequencies [26,27]. A hybrid radar-camera sensing system is designed in [28] to record the random body movement, which is used as a-priori information for cancelling the body movement. As a result, body movement compensation techniques presented so far are rather cumbersome in terms of practical implementations. Consequently, a vital-sign estimation technique that is robust to body movements would be highly appreciable.

In this paper, we investigate the estimation of human vital signs using signals received from an IQ demodulator-based 60 GHz Doppler radar (operating at millimeter-wave frequencies enabled using highly directive antennas, that are useful to spatially discriminate multiple persons in a room, for instance). In particular, we propose an automatic breathing and heartbeat rate detection. The goal is not to provide a waveform to be interpreted by a practitioner, but quantified data that could be used in an autonomous device in order to monitor the elderly at home and detect emergency situations. For that purpose, we propose an optimization procedure based on a direct model describing the human movements. The minimization of a suitable cost function leads then to a robust estimation of the vital signs. This approach depends therefore on the relevance of the direct model. Consequently, this paper presents a thorough analysis of the different parameters involved in the direct model. In particular, to our knowledge, the influence of mutual phasing between the physiological movements on the detection has not been investigated and even not considered in previous studies, especially in the presence of ambiguity. We show that this effect must be taken into account for correct estimations. Furthermore, no statistical analysis has been previously given for comparing different spectrum-estimation algorithms, with diverse physiological possibilities (normal case, no breath, random body motion, etc.), whereas this has a deep influence on the accuracy achieved by each technique. Our proposed approach will be shown to be robust in most scenarios, including when random body movements are present. This paper is organized as follows: The importance of taking into account the mutual phasing is theoretically analyzed in Section 2. The weakness of the direct spectrum analysis is discussed in Section 3. Then, a bound-constrained optimization algorithm is proposed in Section 4 for achieving both accurate respiratory- and heartbeat-rate detection. Results are then statistically compared and shown in terms of a cumulative distribution function (CDF). In Section 5, a parallel optimization is proposed to possibly decrease estimation time while maintaining high accuracy in case of large-scale constrained bound. Different levels of noise are tested and a random body motion is considered as a perturbation in our simulation. In addition, an experimental setup is developed and measurement results are shown and discussed for different configurations. Finally, a conclusion is drawn in Section 6.

## 2. Nonlinearity in Doppler Radar Vital-Signal Detection

**Figure 1:**
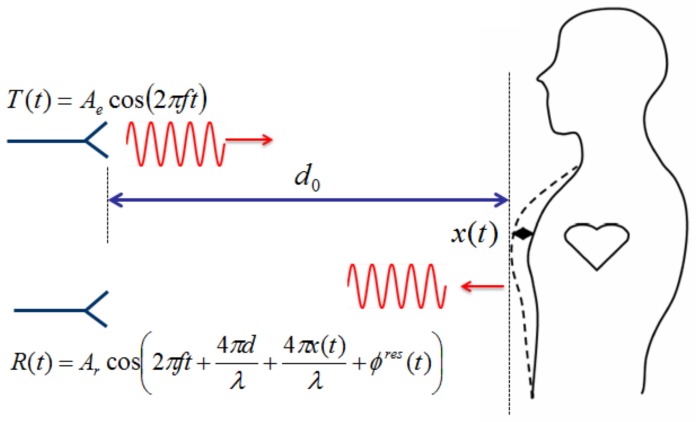
Representation of phase-modulated Doppler radar system by movements of a human body.

In a continuous wave (CW) Doppler radar vital-sign detection system, a sinusoidal signal $T\left(t\right)=A_{e}\cos\left(2\pi ft\right)$ at carrier frequency *f* is transmitted towards a human body, located at a certain distance $d_{0}$, as shown in Figure 1. The signal is reflected by the chest, whose movement x(t) is due to both heartbeating and respiration [29,30,31]. The reflected signal R(t) is demodulated by an IQ quadrature receiver to avoid null-point detection issues [13]. The two baseband signals $B_{I}$ and $B_{Q}$ are of the form

(1) $$\begin{array}{c} B_{I}\left(t\right)=A_{r}\cos\left[\frac{4\pi x(t)}{\lambda }+\frac{4\pi d_{0}}{\lambda }+\theta \left(t\right)\right],\\ B_{Q}\left(t\right)=A_{r}\sin\left[\frac{4\pi x(t)}{\lambda }+\frac{4\pi d_{0}}{\lambda }+\theta \left(t\right)\right], \end{array}$$

and are modulated by physiological movements x(t) of the human body. θ(t) is defined as total residual phase of the radar system. λ=5 mm is the wavelength at f=60 GHz. The physiological movements are represented by the sum of two single-tone sinusoidal signals, $x\left(t\right)=x_{r}\left(t\right)+x_{h}\left(t\right)=m_{r}\sin\left(2\pi f_{r}t+\phi_{r}\right)+m_{h}\sin\left(2\pi f_{h}t+\phi_{h}\right)$. $m_{r}$ and $m_{h}$ describe the movement amplitude of respiration and heartbeat, respectively, $f_{r}$ and $f_{h}$ represent the rate of movement, and $\phi_{r}$ and $\phi_{h}$ are the initial phases for each movement. Typical values for $m_{h}$ lie in the range of 0.08–0.4 mm for an adult, according to measurements with a CCD laser-displacement sensor [32], and $m_{r}$ varies from 0.8 to 6.0 mm if the detection is done in front of the human body, and is about 0.2 mm from the back [33]. The maximum heart rate for a person older than 40 years is about 180 bpm (beats per minute) after exercise, and the average resting rate is between 60–100 bpm [34]. The respiratory rate at rest ranges from 16 to 25 bpm (breaths per minute) [35], and may raise up to 40–50 bpm after exercise [36].

### 2.1. Arctangent Demodulation

As shown in (1), the two baseband signals $B_{I}$ and $B_{Q}$ have a π/2 phase difference, so the total Doppler phase shift can be obtained by computing $\arctan\left[B_{Q}\left(t\right)/B_{I}\left(t\right)\right]=\frac{4\pi x(t)}{\lambda }+\frac{4\pi d_{0}}{\lambda }+\theta \left(t\right)$. The residual phase $\psi \left(t\right)=\theta \left(t\right)+\frac{4\pi d_{0}}{\lambda }$ is assumed to be constant during the observation time, for a still human body. In an ideal case, the Fourier transform of this demodulated signal can directly give the spectral information of the two movements, where the amplitude is inversely proportional to the wavelength. Shorter wavelengths in the denominator provide a higher sensitivity to distinguish small displacements [10,12].

### 2.2. Complex Demodulation

Another technique is the complex demodulation, where the baseband signal is constructed as:

(2) $$\begin{array}{c} B\left(t\right)=B_{I}\left(t\right)+jB_{Q}\left(t\right)=\exp\left[j\frac{4\pi x(t)}{\lambda }\right]\exp\left(j\psi \right). \end{array}$$

By replacing x(t) in (2) by $\left[x_{h}\left(t\right)+x_{r}\left(t\right)\right]$, we obtain the following formula:

(3) $$\begin{array}{c} B\left(t\right)=\exp\left[j\frac{4\pi m_{h}\sin\left(\omega_{h}t+\phi_{h}\right)}{\lambda }\right]\exp\left[j\frac{4\pi m_{r}\sin\left(\omega_{r}t+\phi_{r}\right)}{\lambda }\right]\exp\left(j\psi \right), \end{array}$$

where the first two exponential terms can be expanded using Fourier series [12] as,

(4) $$\begin{array}{c} \exp\left[j\frac{4\pi m_{h}\sin\left(\omega_{h}t+\phi_{h}\right)}{\lambda }\right]=\sum_{n=-\infty }^{+\infty }J_{n}\left(\frac{4\pi m_{h}}{\lambda }\right)\exp\left[j\left(n\omega_{h}t+n\phi_{h}\right)\right], \end{array}$$

and

(5) $$\begin{array}{c} \exp\left[j\frac{4\pi m_{r}\sin\left(\omega_{r}t+\phi_{r}\right)}{\lambda }\right]=\sum_{k=-\infty }^{+\infty }J_{k}\left(\frac{4\pi m_{r}}{\lambda }\right)\exp\left[j\left(k\omega_{r}t+k\phi_{r}\right)\right], \end{array}$$

where $J_{n}$ is the *n*-th order Bessel function of the first kind. Then, (3) can be expressed as

(6) $$\begin{array}{c} B\left(t\right)=\sum_{n=-\infty }^{+\infty }\sum_{k=-\infty }^{+\infty }J_{n}\left(\frac{4\pi m_{h}}{\lambda }\right)J_{k}\left(\frac{4\pi m_{r}}{\lambda }\right)\\ \exp\left[j\left(n\omega_{h}t+k\omega_{r}t\right)\right]\exp\left[j\left(n\phi_{h}+k\phi_{r}\right)\right]\exp\left(j\psi \right). \end{array}$$

The negative frequency components can be eliminated by applying $J_{-n}\left(x\right)={\left(-1\right)}^{n}J_{n}\left(x\right)$. The dc component $J_{0}\left(\frac{4\pi m_{h}}{\lambda }\right)J_{0}\left(\frac{4\pi m_{r}}{\lambda }\right)\exp\left(j\psi \right)$ is negligible for the detection. Then, this baseband signal is represented by a sum of harmonic components $nf_{h}+kf_{r},n=0,1,2,\cdots ,k=0,1,2,\cdots$, where the nonlinear property causes not only the undesired effect of harmonic interference for each physiological movement signal, but also intermodulation effects between these two movements. For example, regarding the former phenomenon, at the desired heartbeating rate $f=f_{h}$ in the spectrum (n=1 and k=0), the corresponding amplitude $J_{1}\left(\frac{4\pi m_{h}}{\lambda }\right)J_{0}\left(\frac{4\pi m_{r}}{\lambda }\right)$ is determined by both $m_{h}$ and $m_{r}$ at the same time. Moreover, regarding the latter effect, we remark that the initial phases $\phi_{r}$ and $\phi_{h}$ have also influences on the spectrum in presence of the ambiguity. In the case that one harmonic component of respiration is equal to, or very close to, the fundamental heartbeat frequency (i.e., $\omega^{\prime }=n^{\prime }\omega_{r}\approx k^{\prime }\omega_{h}$), the Fourier spectrum at $\omega^{\prime }$ is calculated as

(7) $$\begin{array}{c} \left|B(\omega^{\prime })\right|=|J_{n^{\prime }}\left(\frac{4\pi m_{h}}{\lambda }\right)J_{0}\left(\frac{4\pi m_{r}}{\lambda }\right)\exp\left(jn^{\prime }\phi_{h}+j\psi \right)\\ +J_{0}\left(\frac{4\pi m_{h}}{\lambda }\right)J_{k^{\prime }}\left(\frac{4\pi m_{r}}{\lambda }\right)\exp\left(jk^{\prime }\phi_{r}+j\psi \right)|, \end{array}$$

whose amplitude is a superposition of the spectra of each movement, which depends not only on $m_{r}$ and $m_{h}$, but also on $\phi_{r}$ and $\phi_{h}$. This influence makes the accurate rate detection more difficult, as numerically illustrated in the next section.

## 3. Numerical Spectrum Analysis

In this section, simulation results are presented in order to highlight difficulties of the direct spectrum analysis in retrieving vital-sign parameters, in particular, in the presence of ambiguity and perturbations.

### 3.1. Without Noise

**Figure 2:**
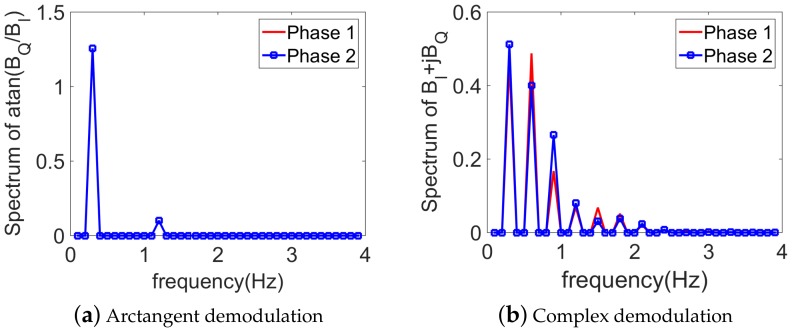
Spectral representation of the noiseless baseband signals, using (**a**) arctangent demodulation and (**b**) complex demodulation. $m_{r}=1.0$ mm, $m_{h}=0.08$ mm. $f_{h}=4f_{r}=72$ bpm (i.e., ambiguity). Red line and blue square line represent different mutual phases ($\phi_{r}$ and $\phi_{h}$, respectively).

In this simulation, the two channels’ baseband signals are in the form of (1). $m_{r}$ is set to 1.0 mm, and $m_{h}=0.08$ mm, corresponding to possible displacements in case of a frontal detection. Note that this case is more difficult than the detection from the back of the body, as the heartbeat strength is much weaker than the respiration. The three phases $\phi_{r}$, $\phi_{h}$, and ψ take arbitrary values uniformly distributed in [0, 2π]. $f_{r}$ is set to 18 bpm, and $f_{h}=72$ bpm, being equal to the fourth harmonic of respiration. An ambiguity is thus present. The observation time window is chosen as T=10 s, and the sampling frequency is $F_{s}=100$ Hz, so that the frequency resolution is Δf=1/T=0.1 Hz. Noiseless simulations show that the arctangent demodulation is more convenient than the complex demodulation in this case, see Figure 2a,b. Only two peaks are present in the arctangent spectrum, corresponding to the frequency component of the respiration and heartbeat, respectively. The peak detection becomes more complicated with the complex demodulation due to the presence of many peaks in the spectrum. The respiratory rate can be determined from the first peak in the spectrum, but the heartbeat rate is not so evident as its frequency component is intermodulated by the harmonics of the respiration. Moreover, the values for $\phi_{r}$ and $\phi_{h}$ have no influence on the arctangent-based spectrum, as shown in Figure 2a. On the other hand, with the complex demodulation, different mutual phases lead to different amplitudes, as shown in Figure 2b. This shift numerically verifies the influence of $\phi_{r}$ and $\phi_{h}$ on the spectrum, as shown in (7), thus making the simple peak detection not reliable. It has to be noted that if the ambiguity is not present, the spectrum of complex demodulation does not depend on $\phi_{r}$ and $\phi_{h}$. The detection is therefore simpler since the amplitudes are related only to $m_{r}$ and $m_{h}$, which can be deduced by taking into account all harmonic components [12].

### 3.2. With Noise

**Figure 3:**
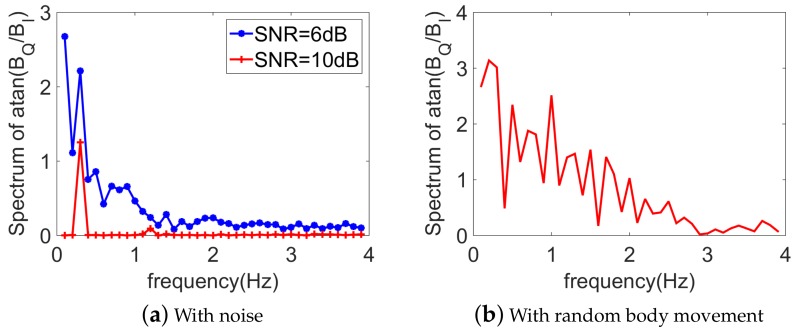
Spectral representation of the baseband signals, using the arctangent demodulation technique. $m_{r}=1.0$ mm, $m_{h}=0.08$ mm. $f_{h}=4f_{r}=72$ bpm (i.e., ambiguity).

As seen in the previous paragraph, without noise, the arctangent demodulation is a straightforward technique as no intermodulation effect takes place. To investigate the robustness of the peak detection with respect to the noise, a zero-mean white Gaussian noise for different signal-to-noise ratios (SNRs) is added to the baseband signal. From Figure 3a, we can see that with a weak noise (e.g., SNR = 10 dB), the direct spectrum analysis of arctangent demodulation succeeds in retrieving two peaks associated to the heartbeat and respiration component, respectively. However, once the noise becomes larger (SNR = 6 dB), this technique does not work as the weak heartbeat signal is buried within the noise. Even without any noise, while an artifact of random body motion is present (which is represented by only half a cycle of a sine wave, with an amplitude 2 cm, a period 0.5 s, and occurs every 5 s), the arctangent demodulation is totally overwhelmed as all peaks in the spectrum are of the same order, as shown in Figure 3b.

### 3.3. Choice of the Demodulation Technique

As seen in the previous paragraph, while the arctangent demodulation technique is straightforward, it fails when the noise becomes strong or in the presence of a random body movement. The complex demodulation, however, is more robust to the noise as shown in the next section, but simple peak detection is not reliable in the presence of ambiguity. To our best knowledge, this ambiguity problem has not been highlighted in the literature and is yet a recurrent event. According to typical scenarios, the 4th breathing harmonic is often close to the heartbeat fundamental. Moreover, in these situations, $\phi_{r}$ and $\phi_{h}$ (which are usually not considered in the literature) have a huge influence on the system response. Consequently, the estimation technique proposed in Section 4 handles these issues.

## 4. Vital-Sign Detection Using Optimization Algorithms

To achieve accurate vital-sign detection with the complex demodulation technique, we propose to apply an optimization algorithm [37], instead of the direct peak detection.

### 4.1. Description of the Problem

The optimization procedure consists of the minimization of a defined cost function, which describes the discrepancy between the received signal (measured data) and the estimated one described by the direct signal model (3). The direct signal model represents the physical model describing the explicit relationship between the observed data and the model parameters, including the heartbeat and respiratory rates of interest. The final solution (model parameters) is obtained once the value of the cost function does not evolve any more or is smaller than a threshold value. Many algorithms exist to solve this problem. One of the most popular techniques is the least-square minimization (LSM), which is more suitable when there are more equations than unknown variables, and the initial estimate needs to be given in advance. Another technique is the genetic algorithm (GA), which was invented by John Holland in the 1960s. There is no need to define the initial estimate as the evolution starts from a population of randomly generated individuals. Another heuristic search method is particle swarm optimization (PSO), developed by Dr. Eberhart and Dr. Kennedy in 1995 [38].

The minimization of the cost function can be performed either in the time domain, or in the frequency domain, and is defined as

(8) $$F\left(\bar{X}\right)=\frac{∥B^{\mathrm{mes}}-B^{\mathrm{est}}\left(\bar{X}\right){∥}^{2}}{∥B^{\mathrm{mes}}{∥}^{2}},$$

where in a real scenario, $B^{\mathrm{mes}}$ refers to the measured baseband signal after IQ complex demodulation (in frequency or time domain). In simulation, $B^{\mathrm{mes}}$ is obtained using (6) by randomly choosing the different parameters involved and adding or not adding a white Gaussian noise, as investigated in the next sections. $B^{\mathrm{est}}$ is the reconstructed signal based on the direct model in (6) and the set of unknown parameters to be estimated: $\bar{X}=\left[m_{r}^{\mathrm{est}}\;m_{h}^{\mathrm{est}}\;f_{r}^{\mathrm{est}}\;f_{h}^{\mathrm{est}}\;\phi_{r}^{\mathrm{est}}\;\phi_{h}^{\mathrm{est}}\;\psi^{\mathrm{est}}\right]$. The goal of the optimization procedure is to find $\bar{X}$ by minimizing the cost function (8) with $\bar{X}$ as variables. The ranges of all variables are defined as lower lb(i) and upper bounds ub(i),

(9) $$lb\left(i\right)\leq {\bar{X}}_{i}\leq ub\left(i\right).$$

The bounds for $m_{r}^{\mathrm{est}},m_{h}^{\mathrm{est}},f_{r}^{\mathrm{est}}$, and $f_{h}^{\mathrm{est}}$ are taken from the typical ranges for a person at rest: [0.1, 1.5] mm, [0.05, 0.15] mm, [12, 25] bpm and [60, 100] bpm, respectively. The bounds for $\phi_{r}^{\mathrm{est}}$, $\phi_{h}^{\mathrm{est}}$, and $\psi^{\mathrm{est}}$ are randomly taken between 0 and 2π. In the time domain, $B^{\mathrm{est}}\left(\bar{X}\right)$ is given by (3), and is consequently defined as the Fourier transform of (3) in the frequency domain. To quantify the quality of the optimization, the estimation error for each variable ${\bar{X}}_{i}$ is defined as

(10) $${\mathrm{Err}}_{{\bar{X}}_{i}}=\frac{|{\bar{X}}_{i}^{\mathrm{actual}}-{\bar{X}}_{i}^{\mathrm{est}}|}{|{\bar{X}}_{i}^{\mathrm{actual}}|}\times 100\%,$$

where ${\bar{X}}_{i}^{\mathrm{actual}}$ is the actual value of ${\bar{X}}_{i}$ while ${\bar{X}}_{i}^{\mathrm{est}}$ is the value estimated by the optimization.

### 4.2. Numerical Results

#### 4.2.1. Without Noise, with Ambiguity

**Figure 4:**
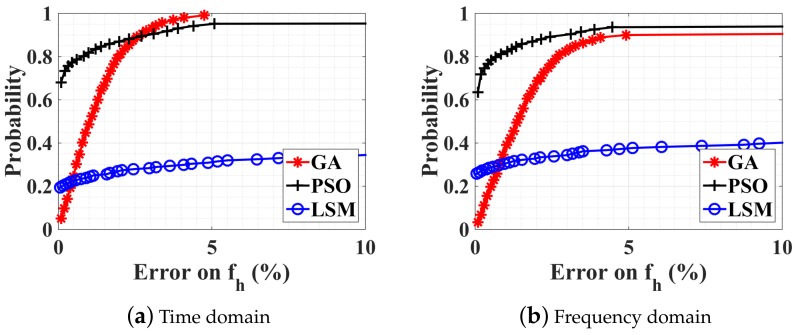
Obtained CDF with optimization in (**a**) time domain and (**b**) frequency domain. Three optimization algorithms are compared (namely, GA, PSO, and LSM). Without noise. $m_{r}=1.0$ mm, $m_{h}=0.08$ mm, and $f_{h}=4f_{r}=72$ bpm (i.e., ambiguity).

Firstly, the feasibility of optimization algorithms is tested using noiseless data, and $f_{h}=4f_{r}=72$ bpm (i.e., in presence of ambiguity). To compare the performance of different methods, simulations have been performed in MATLAB environment on a computer with a 3.6 GHz Intel Core CPU and 32 GB RAM. The CDF is obtained by executing 1000 optimizations. At each iteration, $\phi_{r}$, $\phi_{h}$, and ψ take random values, while $f_{r}$, $f_{h}$, $m_{r}$, and $m_{h}$ are fixed and are the same as in the previous section. The observation time window is chosen to be 10 s. CDFs obtained from the three optimization algorithms (GA, PSO, LSM) in the time and frequency domains are plotted in Figure 4a,b respectively. These results represent the probability that the accuracy of the estimated quantity is greater than a threshold given by the abscissa values. Only the CDF of the estimation error on $f_{h}$ is given here, as the estimation on $f_{r}$ is much easier and always accurate. It can be seen that neither in the time domain, nor in the frequency domain, does the LSM work well. The probability that the error on $f_{h}$ is less than 10% is about 0.35. This highlights the weakness of LSM for this problem, which is very sensitive to the initial estimate. Wrong starting values can cause the cost function to converge to a local minimum rather than the global one that defines the least-squares estimates. It is very troublesome to give an adequate starting point as the seven unknown parameters are independent. The population size $N_{\mathrm{GA}}$ (swarm size $N_{\mathrm{PSO}}$) for the GA (PSO) is 200 and the tolerance value of the cost function is $10^{-3}$. The GA works perfectly in the time domain while a little less accurately in the frequency domain. The PSO is stable as the probability is always converging to 0.95 whatever the working domain, but converges more quickly in the frequency domain (average estimation time 2.6 s) than in the time domain (average estimation time 4.6 s). The average estimation time for the GA is about 3.6 s in the frequency domain and 3 s in the time domain. Note that none of these procedures can converge to 100% as sometimes the local minimum persists. It should be noted that the estimation results in the case of no ambiguity are not shown here but exhibit similar behavior to those with ambiguity.

#### 4.2.2. Noise Influence on the Optimization

**Figure 5:**
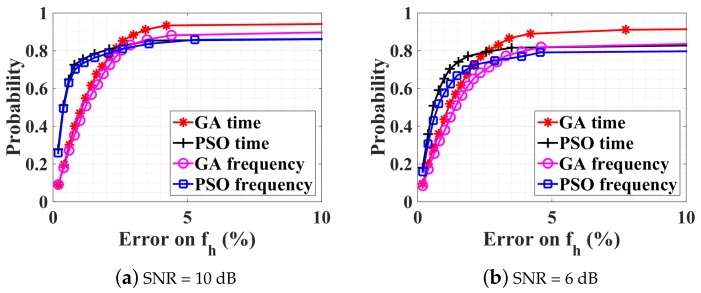
CDFs of different optimization procedures with SNRs at the receiver of (**a**) 10 dB and (**b**) 6 dB. $m_{r}=1.0$ mm, $m_{h}=0.08$ mm, and $f_{h}=4f_{r}=72$ bpm (i.e., ambiguity).

The performance of the GA and PSO algorithms are now investigated in the presence of noise at the receiver. The estimation results are given in Figure 5a,b, for SNRs of 10 dB and 6 dB, respectively. The estimation time is similar to the previous noiseless case. The GA in the time domain works best as the estimation error on $f_{h}$ is less than 10% with a probability of 0.95 (0.90) for SNR =10 dB (6 dB). The other three optimization procedures perform pretty well but are less accurate than the GA in the time domain.

#### 4.2.3. Observation-Time Influence on the Optimization

**Figure 6:**
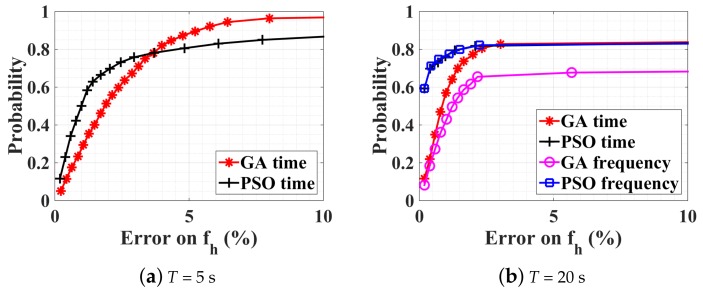
CDFs of different optimization procedures for obervation time duration of (**a**) 5 s and (**b**) 20 s. $m_{r}=1.0$ mm, $m_{h}=0.08$ mm, $f_{h}=4f_{r}=72$ bpm (i.e., ambiguity), and SNR = 10 dB.

Maintaining an SNR of 10 dB, the observation time duration is investigated by using two additional windows of 5 s and 20 s, whose results are shown in Figure 6a,b respectively. For an observation duration of 5 s, the corresponding frequency resolution is 0.2 Hz, which sets the best achievable accuracy. This is not well suited for the considered problem, as the optimization in the time domain under the same condition is always more accurate than 0.2 Hz. From Figure 5a and Figure 6a, it is noticeable that for both GA and PSO, results are more accurate with T=5 s than with T=10 s with an estimation error less than 10%. Moreover, the estimation time is much less: only 1.4 s for GA and 2.2 s for PSO. However, for more accurate results (e.g., less than 2%), T=10 s gives higher probability where the CDF curve is steeper.

Figure 6b shows results for T=20 s. The estimation time for GA is increased to 5.5 s (4.4 s) in the frequency domain (time domain), and to 4.3 s (7.5 s) in the frequency domain (time domain) for PSO. In particular, for frequency-domain GA, performance quickly decreases as the time-window length increases. Comparing the optimization result in the time domain for T=5 s and T=20 s, as shown in Figure 6a,b, with a probability of 80%, the estimation error is less than 4% with GA or PSO for T=5 s, and less than 2% with PSO for T=20 s. However, beyond a certain error on $f_{h}$, we observe that with 20 s, the probability does not increase any more, whereas it does with a 5 s window. Further simulations with different time windows show that, if the window size increases, the precision can actually be improved if a suitable threshold is chosen on the cost function to truncate the optimization process. If the threshold is kept constant, a saturation effect is indeed observed. In these simulations, the parameter $f_{h}$ is kept constant during the observation window (whether it is 5 or 20 s). In a real scenario, breathing and heartbeat rate will be time dependent. Since the accuracy reached is considered satisfying for medical applications and a constraint on the computation time is preferred in this work for real-time applications, this phenomenon is not further illustrated here. Consequently, the next section investigates the robustness of these techniques in a broader range of scenarios.

## 5. Large-Scale Constrained Bound: PSO Parallel Optimization

In Section 4.2.1, values of $m_{r}$, $m_{h}$, $f_{r}$, and $f_{h}$ were fixed all the time, and the range of the constrained bound was limited to normal situations. In practice, these values depend on individual physiology and would vary widely from person to person. In order to test the robustness of our optimization algorithm in diverse situations, we propose to investigate different scenarios. The PSO method will be shown to be the most effective approach to estimate vital signs in a wide range of physical conditions.

### 5.1. Normal Case

The amplitude $m_{r}$ is firstly increased to 2 mm, and $m_{h}$ to 0.3 mm, which correspond to RMS (root mean square) motion values indicated in [33]. This case is more delicate than the previous one since $m_{r}$ is close to 0.3λ at 60 GHz, yielding stronger interference effects as high-order Bessel functions $J_{n}$ become more important in the spectrum [39]. The intermodulation between the heartbeat and the respiration is also more prominent as the ratio of $m_{h}/m_{r}$ is 0.15 with respect to 0.08 for the previous case. $f_{r}$ takes a random value between [12, 25] bpm, and $f_{h}$ varies between [60, 100] bpm, which correspond to a normal adult respiratory and heartbeat rate. The scale of constrained bound is enlarged to upper and lower possible physiological limits in Table 1, including both the possibilities of “at rest” and “after sport” conditions. Note that the lower limit for $m_{r}$ is set to 0 where a no-breath case is also considered. As the range of the parameters to optimize is larger, a larger number of initial populations $N_{\mathrm{GA}}$ is needed in the GA optimization. This is illustrated in the following case, where $N_{\mathrm{GA}}=500$ and $N_{\mathrm{PSO}}=50$. In order to maximize the accuracy, the following procedure has been adopted. Independent optimizations are executed in parallel, each one having its own randomly chosen initial populations. The procedure is stopped once one value of the cost functions reaches a threshold. It has been verified that a further increasing of $N_{\mathrm{GA}}$ or $N_{\mathrm{PSO}}$ does not improve the convergence but requires more estimation time. The threshold value of the normalized cost function is set to 0.2, which corresponds approximately to a 20% estimation error. It is emphasized that this value could be adjusted upon different experimental conditions, for example, with stronger measured noise, the threshold could be slightly raised.

**Figure 7:**
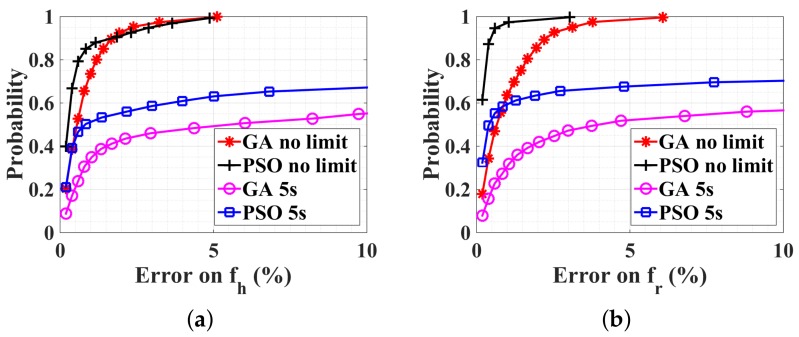
CDFs of different optimization procedures with a large–scale constrained bound. SNR = 10 dB, $m_{r}=2$ mm, $m_{h}=0.3$ mm, $f_{r}=\left[12,25\right]$ bpm, and $f_{h}=\left[60,100\right]$ bpm. (**a**) Estimation error on *f_h_*, (**b**) Estimation error on *f_r_*.

The CDFs of optimization results on $f_{h}$ and $f_{r}$ are shown in Figure 7a,b, respectively. It can be seen that both GA and PSO work perfectly with no limited estimation time, where the estimation error is always less than 10%. However, the average estimation time for GA is 12.9 s (with a standard deviation 10.3 s), and for PSO is 4.5 s (with a standard deviation 7.0 s), which are too time consuming for real-time physiological applications. Thus, if the estimation time is limited to 5 s, corresponding to the blue-square and magenta-circle lines in Figure 7, the estimation quality is deteriorated with respect to the case with narrower ranges (Figure 6a). Due to the large-scale constrained bound, our optimization algorithms can work, but need more estimation time to converge on the good solution.

In order to guarantee the convergence of the optimization with an estimation time less than 5 s, it is proposed to divide each wide range for $f_{r}$ and $f_{h}$ into two subranges, respectively. Each subrange corresponds to the bounds of rest case and after-sport case. Then, four possible subregions are generated, including also the cross possibilities, as shown in Table 1. The ranges for $m_{r}$ and $m_{h}$ are not modified. The optimization is executed individually in each subrange. Once the value of the cost function of one subrange converges to 0.2, all other subrange optimizations are stopped. Alternatively, if the estimation time exceeds 5 s, all optimization procedures are abandoned. The subrange having a minimum final value of the cost function is retained as the optimal solution. Only the PSO algorithm is used as it is more efficient than GA, as shown in Figure 7.

**Figure 8:**
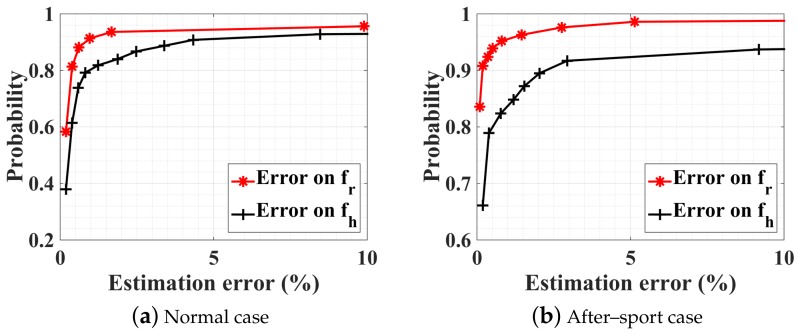
CDFs of PSO optimization procedure executed in four sub-bounds for (**a**) normal case: $f_{r}=\left[12,25\right]$ bpm, $f_{h}=\left[60,100\right]$ bpm, and (**b**) rapid case: $f_{r}=\left[25,72\right]$ bpm, $f_{h}=\left[100,180\right]$ bpm. SNR = 10 dB. $m_{r}=2$ mm, $m_{h}=0.3$ mm.

Optimization results are shown in Figure 8a, and exhibit the same performance as the case of small scale, in Figure 6a. The average estimation time for the convergence of the cost function is only 1.7 s (with a standard deviation 1.4 s), that is, about one-quarter of the single large-scale case. This parallel optimization works also very well with rapid breath and heart rate, for example, after doing sport, as shown in Figure 8b.

### 5.2. No-Breath Case

**Figure 9:**
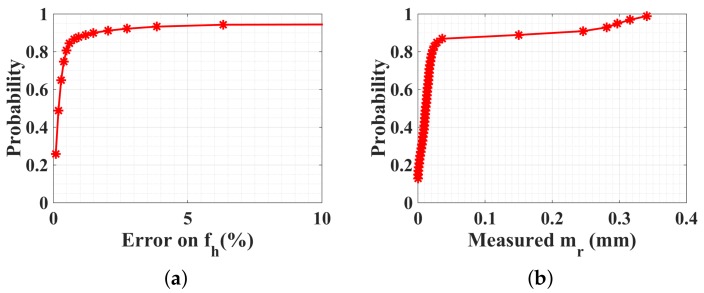
CDFs of PSO optimization procedure executed in four subranges. The person under test does not breathe but has a normal heart rate. $m_{r}=0$ mm, $m_{h}=0.3$ mm, and $f_{h}=\left[60,100\right]$ bpm. SNR = 10 dB.

Here, a particular case is considered where the person does not breathe, that is, $m_{r}=0$ mm. The cardiac rhythm is supposed to be normal, taking a random value $f_{h}=\left[60,100\right]$ bpm. The optimization result on $f_{h}$ is always accurate, as shown in Figure 9a, where the probability is 0.95 for the estimation error less than 10%. The absolute estimated amplitude of respiratory displacement $m_{r}^{\mathrm{est}}$ is given in Figure 9b, which is always less than 0.2 mm with a probability of 0.9. Such a weak estimated value shows that the human body does not breathe any more or his respiration is very weak, which may indicate an emergency situation.

### 5.3. With a Random Body Motion

As a final test, $f_{r}$, $f_{h}$, $m_{r}$, and $m_{h}$ take random values generated from a Gaussian distribution, within ranges in Table 1. Moreover, the same random body movement in Figure 3b is plugged into the reflected baseband signal. The threshold value of the cost function is increased to 0.3, as several perturbations exist. The optimization time is increased to 10 s as it is verified that 5 s is not enough to get an accurate detection in the presence of random body motion.

**Figure 10:**
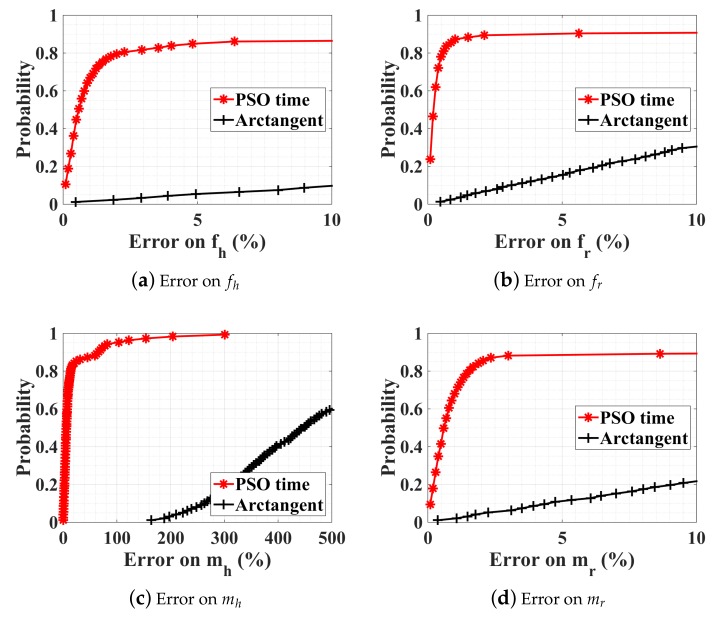
CDFs of PSO optimization procedure in four subranges and arctangent direct peak detection. SNR = 10 dB, and with a random body motion. $m_{r}$, $m_{h}$, $f_{r}$, and $f_{h}$ take random values within ranges indicated in Table 1. Results are obtained for 1000 generations.

The PSO estimation result is compared with the arctangent direct peak detection, in Figure 10. As already discussed in Section 3.2 (Figure 3b), the direct peak detection does not work due to the random body motion. The PSO parallel optimization succeeds not only in the estimation of breathing and heartbeat rate, but also in the estimation of the amplitude of displacement, which can be used as additional information for health monitoring. In order to summarize all the different detection methods proposed in the paper, the approaches, together with the relevant advantages and disadvantages, are given in Table 2.

### 5.4. Experimental Measurements

**Figure 11:**
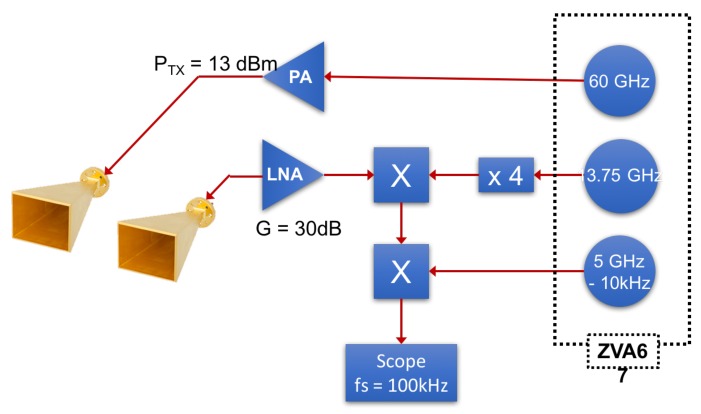
Experimental assemblage of 60 GHz Doppler radar system.

**Figure 12:**
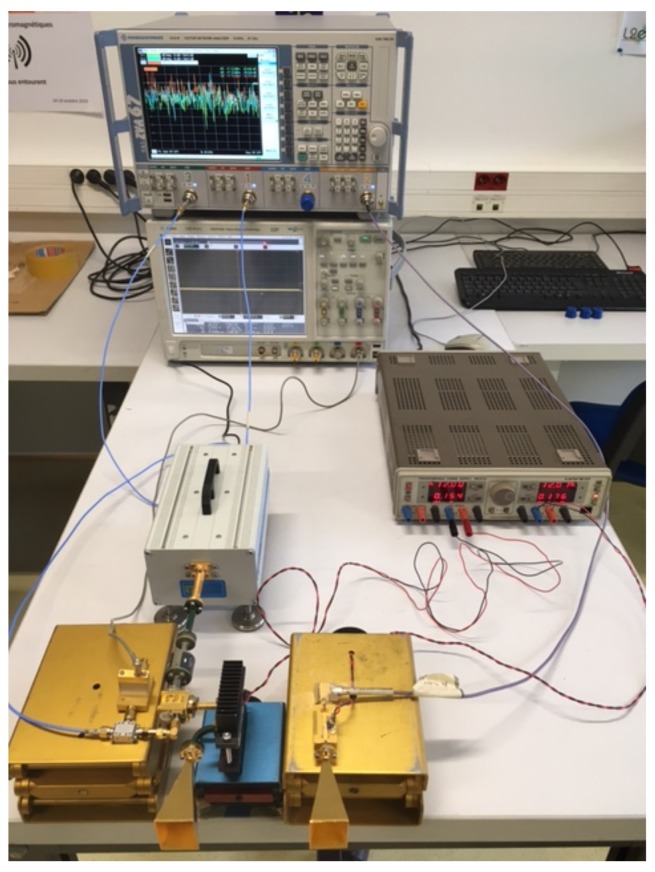
Photo of experimental setup of 60 GHz Doppler radar system.

In order to assess the performance of the optimization technique with respect to the arctangent demodulation on the estimation of real data, a 60 GHz noncontact Doppler radar system is developed using the configuration presented in Figure 11. Both transmitter and receiver use a horizontally polarized horn antenna. A 60 GHz wave is transmitted and the reflected wave is then down-converted using two mixers to a signal at intermediate frequency of 10 kHz, which is sampled at $f_{s}=100$ kHz with an oscilloscope. The recorded signal is then down-converted to an IQ baseband signal in Matlab, on which the heartbeat rate estimation is performed. The three local oscillators at the frequencies 60 GHz, 13.75 GHz, and 5 GHz (see Figure 11), are provided by a Rodhe & Schwarz ZVA67 and do not exhibit phase drift between each other. A photo of the setup is given in Figure 12. The antennas are directed to the chest of an adult at a distance of 2 m.

**Figure 13:**
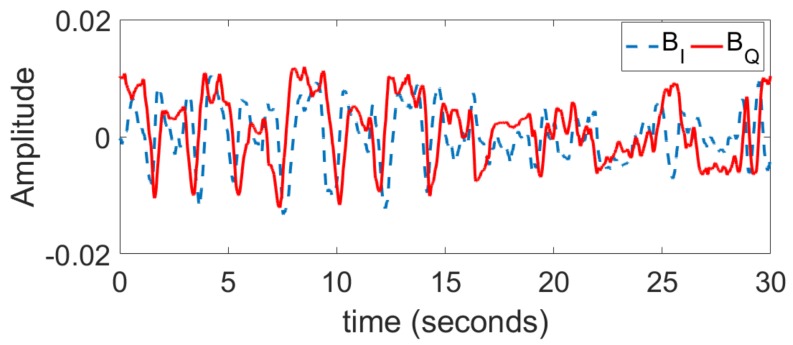
Measured demodulated IQ signal when the person under test is at rest.

**Figure 14:**
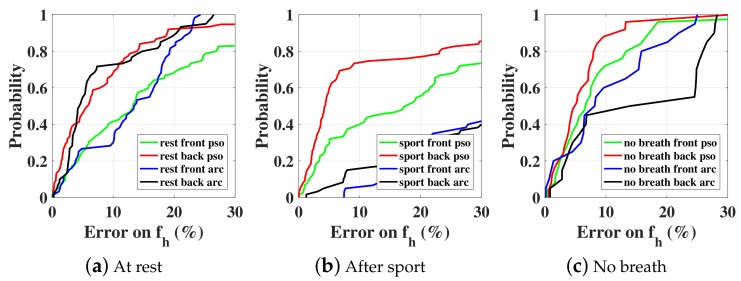
CDFs of PSO optimization procedure in four subranges and arctangent direct peak detection.

Three different scenarios are considered: at rest, after sport, and holding breathing. For each scenario, the vital signal is collected three times during 30 s from the front and the back of the test subject (male, 1.82 m height, 80 kg). The I and Q baseband signals for one case at rest are shown in Figure 13. PSO estimation results are compared with the arctangent direct peak detection in Figure 14 regarding the heartbeat rate. The accuracy is calculated by comparing the obtained results with independent measurements given by a pulse oximeter sensor attached to the subject’s little finger. The PSO algorithm is executed each time on a sliding time window (total length 5 s, sliding 1 s each time) and the optimization time is limited to 2 s. As the computation time is less than the observed time, we can state that this is close to real-time monitoring. For now, we record the measured data with an oscilloscope, thus about 3 s is needed for the buffering time. In the future, once the data is recorded and processed on chip, both the buffering and computation time should be saved. All other optimization parameters are identical to the simulation part. As shown in Figure 14, the PSO estimation always leads to a better accuracy than the arctangent peak detection. In particular for the case ‘after sport’ where the person’s chest moves faster and with a greater amplitude, the arctangent estimation is not suitable any more, since the performance is heavily deteriorated compared to the ‘at rest’ case. However, the PSO optimization still exhibits a satisfactorily accuracy. Note that the estimation is always more accurate when the subject is illuminated from the back rather than from the front. This is due to the fact that the amplitude of respiration is smaller, and the intermodulation between both breathing and heartbeat movements is less. Finally, the PSO technique enables the recognition of the ‘no-breath’ event. Indeed, the optimization leads to an estimation of the respiration movement $m_{r}$ in the order of 0.01 mm. Such an amplitude that is close to null can inform that the person is actually not breathing, and can be a useful feature for the detection of emergency.

## 6. Conclusions

In this paper, estimation techniques of vital-sign monitoring based on 60 GHz Doppler radar have been studied. This operating frequency enables higher sensitivity for heartbeat detections but may face stronger intermodulation effects with respiration signals. PSO and GA optimization procedures have been investigated and found to be able to achieve accurate detections, even in the presence of ambiguity and strong intermodulations. In the presence of a large-scale constrained bound, the parallel PSO in the time domain is proved to be the optimal choice. The importance of mutual phasing on the detection has been moreover discussed in detail. The proposed PSO parallel algorithm is further applied on experimental data, where three different scenarios were investigated: ‘at rest’, ‘after sport’, and ‘no breath’. The advantage of PSO with respect to the direct spectrum analysis is consistent in both simulated and measured data.

The proposed optimization procedure has therefore promising applications in autonomous noncontact health monitoring for people at home, in particular for detecting weak heartbeat signals and quantifying induced displacements of the human body. More sophisticated signal processing techniques will be studied for cancelling stronger undesired random body movements in future studies.

## Figures and Tables

**Table 1:** Large-scale constrained bound.

		$f_{r}$ (bpm)	$f_{h}$ (bpm)	$m_{r}$ (mm)	$m_{h}$ (mm)
At rest	lb	12	48	0	0.05
	ub	30	90	6.0	1.0
After sport	lb	30	90	0	0.05
	ub	60	180	6.0	1.0

**Table 2:** Comparison of different estimation methods.

Working Domain	Methods		Advantages	Disadvantages
Frequency domain	Peak detection	Arctangent demodulation	Fast, No ambiguity	Sensitive to noise and to random body movements, Needs accurate DC offset compensation
		Complex demodulation	Fast, Robust to noise	Intermodulation, ambiguity
	Optimization	LSM, GA, and PSO	Handle ambiguity	At least 10 s time window, Not adaptable to nonstationary signal
Time domain	Optimization	LSM	Converge quickly	Sensitive to initial estimates, Easy to fall into local minima
		GA	Robustness, Stable	Computationally expensive if applied to large bounds
		PSO	Converges more quickly than GA	
		PSO in parallel	Robust, Less optimization time	Multiple processors required

## Abbreviations

WSN	Wireless sensor network
IQ	In-phase quadrature
LO	Local oscillator
EEMD	Ensemble empirical mode decomposition
CW	Continuous wave
CDF	Cumulative distribution function
LSM	Least-square minimization
GA	Genetic algorithm
PSO	Particle swarm optimization
